# Transplastomic integration of a cyanobacterial bicarbonate transporter into tobacco chloroplasts

**DOI:** 10.1093/jxb/eru156

**Published:** 2014-04-18

**Authors:** J. J. L. Pengelly, B. Förster, S. von Caemmerer, M. R. Badger, G. D. Price, S. M. Whitney

**Affiliations:** Plant Science Division, Research School of Biology, The Australian National University, Canberra, ACT 0200, Australia

**Keywords:** Bicarbonate pumps, chloroplast envelope, CO2-concentrating mechanism, crop improvement, cyanobacteria, photosynthetic efficiency, plastome transformation.

## Abstract

Improving global yields of agricultural crops is a complex challenge with evidence indicating benefits in productivity are achieved by enhancing photosynthetic carbon assimilation. Towards improving rates of CO_2_ capture within leaf chloroplasts, this study shows the versatility of plastome transformation for expressing the *Synechococcus* PCC7002 BicA bicarbonate transporter within tobacco plastids. Fractionation of chloroplast membranes from transplastomic tob^BicA^ lines showed that ~75% of the BicA localized to the thylakoid membranes and ~25% to the chloroplast envelope. BicA levels were highest in young emerging tob^BicA^ leaves (0.12 μmol m^–2^, ≈7mg m^–2^) accounting for ~0.1% (w/w) of the leaf protein. In these leaves, the molar amount of BicA was 16-fold lower than the abundant thylakoid photosystem II D1 protein (~1.9 μmol m^–2^) which was comparable to the 9:1 molar ratio of D1:BicA measured in air-grown *Synechococcus* PCC7002 cells. The BicA produced had no discernible effect on chloroplast ultrastructure, photosynthetic CO_2_-assimilation rates, carbon isotope discrimination, or growth of the tob^BicA^ plants, implying that the bicarbonate transporter had little or no activity. These findings demonstrate the utility of plastome transformation for targeting bicarbonate transporter proteins into the chloroplast membranes without impeding growth or plastid ultrastructure. This study establishes the span of experimental measurements required to verify heterologous bicarbonate transporter function and location in chloroplasts and underscores the need for more detailed understanding of BicA structure and function to identify solutions for enabling its activation and operation in leaf chloroplasts.

## Introduction

Many important food crops including rice (*Oryza* spp.) and wheat (*Triticum* spp.) utilize the C_3_ photosynthetic pathway. Improving photosynthesis in such species via a range of engineering strategies has been identified as a promising strategy for crop improvement with regard to increased photosynthetic yield and better water use efficiency (von Caemmerer and Evans, 2010; Price *et al.*, 2010; Parry *et al.*, 2013). Many of these strategies pose solutions for improving CO_2_ capture by the enzyme ribulose-1,5-bisphosphate carboxylase/oxygenase (Rubisco) located in the chloroplast stroma. Rubisco catalysis involves reacting CO_2_ with the 5-carbon substrate ribulose-1,5-bisphosphate (RuBP) to produce two 3-phosphoglycerate (3-PGA) molecules, the precursors of carbohydrate synthesis. Although it is the biosphere’s primary CO_2_-fixing enzyme, Rubisco is considered a poor catalyst as it is slow (each enzyme complex catalysing less than three cycles per second in plants) and unable to fully distinguish substrate CO_2_ from the more abundant O_2_. Rubisco oxygenation of RuBP produces 2-phosphoglycolate (2-PG), whose recycling back to 3-PGA by photorespiration results in carbohydrate loss (up to 50% of fixed CO_2_ in some C_3_ plants) and energy consumption. Many cyanobacteria, algae, and land plants have developed active CO_2_-concentrating mechanisms (CCMs) to overcome Rubisco’s inefficiencies (Badger *et al.*, 1998).

In C_3_ plants, photosynthesis is inherently impeded by CO_2_ supply to Rubisco. Here, assimilation is restricted by limitations in passive diffusion of CO_2_ through leaf pores (stomata), across cell walls, plasma membranes, cytosol, and chloroplast membranes, and in the stroma (Evans *et al.*, 2009). The evolution of CCMs has helped circumvent CO_2_-supply limitations by expending energy to pump CO_2_ around Rubisco. In C_4_ plants, the benefits associated with incorporating a CCM are improvements in water, nitrogen, and light use (Sage *et al.*, 2012). As a first step towards introducing a functional CCM into C_3_-plants, Price *et al.* (2013) proposed that introducing single-gene cyanobacterial bicarbonate (HCO_3_^–^) transporters such as BicA (Price *et al.*, 2004) or SbtA (Price *et al.*, 2011) into the envelopes of C_3_ plant chloroplasts may increase CO_2_ supply to Rubisco. By this approach, active transport of HCO_3_^–^ through the chloroplast membrane poses the first step in developing a CCM for increasing stromal CO_2_ partial pressure around Rubisco, thereby reducing oxygenase activity and the costs associated with photorespiration.

BicA is a very suitable target as a first transporter to introduce into the chloroplast because three diverse BicA members of cyanobacterial origin have been shown, by gain-of-function analysis, to be Na^+^-dependent HCO_3_^–^ transporters with high flux rate characteristics (Price *et al.*, 2004). As previously discussed (Price *et al.*, 2010), there are good reasons to conclude that the Na^+^ gradient across the chloroplast envelope and the steady state level of HCO_3_^–^ in the cytoplasm are sufficient for BicA function. Given that the chloroplast is an evolutionary analogue of a cyanobacterial cell, the insertion/folding and orientation of BicA in the inner envelope membrane (IEM) is expected to be governed by its 12 hydrophobic membrane-spanning domains and the preponderance of positively charged amino acid residues on the cytoplasmic face of the transporter, also known as the ‘positive-inside’ rule (Shelden *et al.*, 2012). To maximize function of BicA or SbtA, later engineering enhancements might include downregulating the expression of aquaporins in the IEM (to retard CO_2_ leakage from the chloroplast) and upregulating the key chloroplastic Na^+^/H^+^ antiporter to maximize the Na^+^ gradient (see Price *et al.*, 2013).

The double envelope lipid–bilayer chloroplast membrane represents a significant diffusion limitation to the movement of photosynthetic metabolites, ions and gases into and out of the stroma (Evans *et al.*, 2009). A network of integral and membrane-bound transporter proteins associated with the plastid envelope regulate biochemical exchanges between the stroma and cell cytoplasm (Joyard *et al.*, 1998; Weber and von Caemmerer, 2010). In particular, the IEM houses many transporters that exhibit high substrate selectivity (Linka and Weber, 2010), whereas the outer envelope membrane contains only about 30 different proteins (in *Arabidopsis*), which include proteins of the outer membrane protein import machinery, lipid biosynthetic enzymes, few solute carriers, and proteins involved in organelle division (Inoue, 2011). In vascular plants, the IEM proteins are almost exclusively nucleus encoded (Ferro *et al.*, 2002, 2010), except for the chloroplast genome (plastome) *cem*A gene product (Wicke *et al.*, 2011). While CemA is inferred to indirectly influence inorganic carbon uptake in plastids based on sequence similarities with *Chlamydomonas ycf10* (Rolland *et al.*, 1997) and *Synechococcus* PCC6803 *pxcA* (Sonoda *et al.*, 1998), such a functional role remains unresolved. Nevertheless, the inherent IEM localization of CemA and the proven feasibility of effectively engineering both stromal and membrane heterologous proteins via chloroplast transformation (Verma *et al.*, 2008; Whitney and Sharwood, 2008) supports the practicality of introducing additional proteins into the IEM via genetic modification of the plastome. Already, plastome transformation has been used to successfully integrate the *Arabidopsis* Tic40 chloroplast translocation membrane protein into tobacco chloroplast membranes and to express a functional *Chlamydomonas* plastid terminal oxidase 1 in tobacco thylakoid membranes (Singh *et al.*, 2008; Ahmad *et al.*, 2012). Importantly, expressing heterologous membrane proteins in plastids represents challenges with regard to confirming their correct integration into the IEM, ensuring their biological functionality is maintained, as well as avoiding hyperexpression that can lead to unwanted changes in membrane proliferation and chloroplast ultrastructure (Breuers *et al.*, 2012).

This study demonstrates the feasibility of expressing the *Synechococcus* PCC7002 BicA bicarbonate transporter in tobacco chloroplasts via plastome transformation. It shows that BicA can be expressed in plant chloroplasts, with immunoblot BicA detection of fractionated cellular proteins showing that it localizes to both the chloroplast envelope, most likely the IEM, and thylakoid membranes without apparent detriment to chloroplast ultrastructure. Despite making ample amounts of BicA protein, a notable milestone, no clear evidence for HCO_3_^–^-transporter functionality was detected using measurements of carbon isotopic discrimination and photosynthetic assimilation responses under limiting CO_2_ levels.

## Materials and methods

### BicA vector construction and transformation

**Fig. 1.**
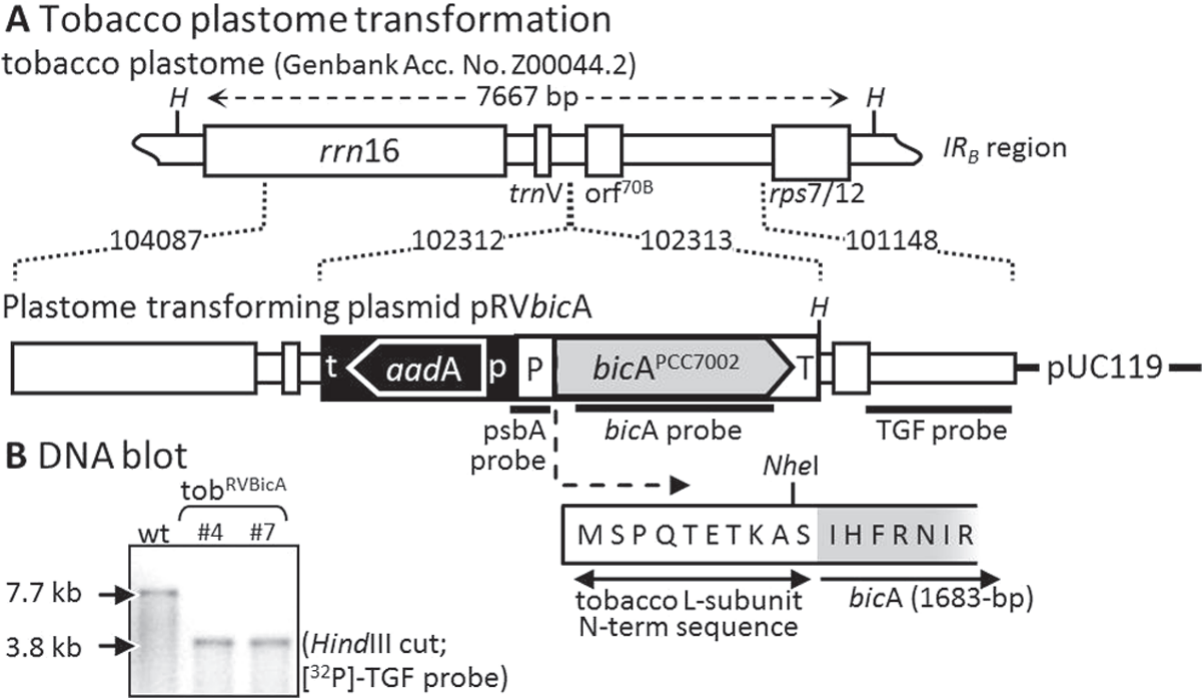
Transformation of *Synechococcus* PCC7002 *bicA* into the inverted repeat (IR) regions of the tobacco plastome. (A) Tobacco plastome sequence in transforming plasmid pRV112a ((Zoubenko *et al.*, 1994); cloned IR_B_ sequence regions are indicated. rps16 3′-UTR (t) and rrn promoter and 5′-UTR (p) directed integration of the selectable marker genes *aadA* and *bic*A by homologous recombination (Whitney and Sharwood, 2008). The tobacco *psbA* promoter/5′-UTR (P) and 114-bp of its 3′-UTR (T) sequence were used to regulate expression of *bicA* modified to code 10 N-terminal residues of the Rubisco L-subunit located 5′ to a unique *Nhe*I cloning site. Correct transformation into the tobacco plastome introduced a unique *Hind*III site (*H*). The annealing positions of nucleotide probes are shown. (B) Total leaf DNA from independently transformed T_1_ tob^BicA^ lines examined by Southern blot analysis. In wild-type plants (wt), the ^32^P-labelled 900-bp TGF probe recognizes a single 7.7-kb fragment and a 3.8-kb fragment in the transformants.

The tobacco plastome-transforming plasmid pRVBicA is a derivative of the plastome-transforming plasmid pRV112a (Zoubenko *et al.*, 1994) containing the *bicA* gene, which was amplified from *Synechococcus* PCC7002 genomic DNA using primers 5′NheIBicA (5′-TTGCTAGCATTCACTTTAGGAATATCC-3′) and 3′XhoIBicA (5′-TTCTCGAGTTAACCCATCTCTGAACTG GG-3′) (Fig. 1A). The codons of the 10 N-terminal amino acids of the Rubisco L-subunit were fused to the 5′ end of *bicA*, and expression was regulated by the tobacco *psbA* promoter/5′-untranscribed region (UTR) and 114bp of the *psbA* 3′-UTR (Whitney *et al.*, 2001). pRVBicA was biolistically transformed into sterile leaves of *Nicotiana tabacum* L. (Petit Havana [*N,N*]) and spectinomycin-resistant plants regenerated in tissue culture as described (Whitney *et al.*, 2009). Following three rounds of regeneration on selective spectinomycin (0.5mg ml^–1^) media, two independent homoplasmic transplastomic lines (B4 and B7) were identified by DNA blotting (Fig. 1B), performed as previously described (Sambrook *et al.*, 1989; Whitney and Andrews, 2001). Both lines were transferred to soil and grown to maturity in growth chambers under a 14/10 light/dark cycle (350±100 μmol quanta m^–1^ s^–2^ illumination, 25/20 °C). Flowers from each generation were fertilized with pollen from nontransformed (wild type) tobacco. Both lines were treated as identical and the B7 line is referred to as tob^BicA^ for this study.

### Plant growth

For all comparative analyses, the T_3_ progeny of transplastomic tob^BicA^ were grown alongside wild-type tobacco controls in 2 l pots of soil under natural illumination in a glasshouse at 25±4 °C. For isolating intact chloroplasts, wild-type and transgenic plants were grown in a chamber under a 16/8 light dark cycle with low irradiance (100 µmol photons m^–2^ s^–1^), at 25 °C and with 65% humidity. These plants were sown as sparse lawns in 30×30cm trays and the juvenile plantlets (with five or six true leaves) were harvested for chloroplast isolation.

### RNA extraction and Northern blotting

Total leaf RNA was extracted from 0.5cm^2^ of different age leaves in the upper canopy of 40±4-cm-tall wild-type and tob^BicA^ plant leaves as described (Whitney *et al.*, 2011). RNA from comparable areas of leaf were separated by denaturing formaldehyde gels and blotted onto nylon membranes and transcripts were detected using ^32^P-labelled DNA probes (Whitney and Andrews, 2001). RNA content was quantified using a nanodrop spectrophotometer (Thermo Scientific). The ^32^P-labelled DNA probes were PCR products of *N. tabacum* plastome sequence (GenBank Z00044.2) spanning 991bp of *rbcL* (nucleotides 57 860–58 850) and 222bp of the *psbA* promoter and 5′ untranslated region (nucleotides 1598–1818). The *bicA* probe comprised 1682bp of the *Synechococcus* PCC7002 genomic *bicA* sequence (nucleotides 2 452 851–2 454 533, GenBank CP000951.1). The ^32^P-signals were digitally detected with a Pharos imager (Biorad) and their densitometry measured using QuantityOne software (BioRad).

### Protein extraction and Western blotting

Total soluble protein was extracted from leaf discs in ice-cold extraction buffer (100mM Tris-HCl pH 8, 10mM MgCl_2_, 10mM NaHCO_3_, 1mM ethylenediaminetetraacetic acid (EDTA), 2mM 1,4-dithiothreitol (DTT), 1% (w/v) polyvinyl polypyrillidone (PVPP), and 1% (v/v) protease inhibitor cocktail (Sigma)) and samples of total and soluble cellular protein taken for PAGE analysis as described (Sharwood *et al.*, 2008). The soluble protein content was measured using the Dye-binding assay (Pierce) against bovine serum albumin standards and Rubisco contents quantified by ^14^C-CABP (carboxyarabinitol 1,5-bisphosphate) binding assays as described (Whitney and Sharwood, 2008). *Synechococcus* PCC7002 cells were cultured as previously described (Price *et al.*, 2004) and harvested during exponential growth by centrifugation (6000 *g*, 25 °C, 4min) and total cellular protein was extracted in an extraction buffer containing 4% (w/v) SDS and no PVPP.

Proteins samples were separated by SDS-PAGE and transferred to nitrocellulose membranes then immunoprobed with primary antibodies raised in rabbits against tobacco Rubisco (Whitney *et al.*, 2001), PsbA (Agrisera), PIP1B (Agrisera), or BicA. A polyclonal BicA-specific antibody had been generated previously against part of the BicA STAS domain (Shelden *et al.*, 2010). This protein domain was cloned into the pET28a expression vector linked to a N-terminal His6-tag, subsequently expressed in *Escherichia coli* BL21/DE3 cells, purified by immobilized metal affinity chromatography and injected into rabbits for antibody generation.

Protein blots were probed with secondary antibody (alkaline phosphatase-conjugated anti-rabbit antibody, BioRad) and the immune-reactive bands detected using Attophos (Promega) and the immunofluorescent signal detected with a VersaDoc imager (Biorad) and the signal intensities quantified using QuantityOne (BioRad).

### BicA and D1 quantitation

BicA and D1 levels in leaf and cyanobacterial cell protein extracts were quantified from Western blots against linear ranges of purified BicA STAS protein domain (19.3kDa sulphate transporter anti-sigma factor antagonist domain, 1–5ng (0.05–0.26 pmol per lane; Shelden *et al.*, 2010) and a 41.5kDa D1 protein (5–41ng (0.12–1.00 pmol per lane; Agrisera). Both protein standards incorporated polyhistidine tags facilitating purification by immobilized metal affinity chromatography. Protein blots to quantify BicA and D1 levels contained both cellular protein extracts and a dilution series of immobilized metal affinity chromatography pure STAS and D1 proteins for constructing standard curves.

### Chloroplast isolation and membrane protein separation

Intact chloroplasts were isolated from lawns of young tobacco leaves using a method derived from Kubis *et al.* (2008). To reduce leaf starch, the plants were kept in darkness for 48h prior to extraction. Approximately 150g of leaf was homogenized with a commercial stick blender (SM6400, Sunbeam) in 100ml of ice-cold isolation buffer (20mM HEPES pH 8, 0.3M sorbitol, 5mM MgCl_2_, 5mM EGTA, 5mM EDTA and 10mM NaHCO_3_) for 1min. The homogenate was filtered through two layers of gauze and Miracloth (Millipore) and centrifuged at 3000 *g* for 5 mins at 4 °C in a Beckman SLA1000 fixed-angle rotor with slow deceleration. The pellet was gently resuspended in 10ml isolation buffer by swirling, then 5ml was carefully layered on top of a phosphate-buffered Percoll 2-step gradient (9ml of 40% and 16ml 80%, v/v, Percoll) containing 10% (w/v) PEG 6000 and centrifuged at 6000 *g* for 20 mins at 4 °C in a Beckman HB4 swinging rotor. The intact chloroplast fraction at the 40 and 80% Percoll interface was recovered, washed twice with 5ml isolation buffer following centrifugation (6000 *g*, 10min, 4 °C) before suspending in ~5ml isolation buffer. Chloroplast purity and intactness was confirmed using a Nikon Eclipse 50i light stereomicroscope and their concentrations quantified with a haemocytometer. To separate the thylakoid from the chloroplast envelope membrane fraction, intact chloroplast fractions were disrupted by five freeze–thaw cycles in quick succession (liquid nitrogen and 37 °C water bath) followed by homogenization with 0.1mm zirconia/silica beads (Daintree Scientific) in ~12ml hypertonic solution (10mM HEPES pH 7.8, 4mM MgCl_2_). Aliquots (~5ml) of the broken chloroplast suspensions were loaded onto three-step sucrose gradients (4ml each of 0.3, 0.6, 0.93M sucrose in hypertonic buffer) and centrifuged at 113,000 *g* for 3h at 4 °C in a Beckman SW32.1 rotor. Envelope membrane fractions were collected from both sucrose interfaces and the thylakoid fraction formed the pellet. The membrane fractions were solubilized in protein extraction buffer (100mM Tris-HCl pH 7.8, 25mM NaCl, 20mM EDTA, 2% (w/v) SDS, 10mM DTT, 5% (v/v) glycerol, 1% (v/v) protease inhibitor cocktail) and aliquots were separated by SDS-PAGE on 4–12% Bis-Tris gels (Invitrogen). Confirmation of chloroplast envelope and thylakoid membrane purity was achieved by immunoblotting with the envelope-specific anti-PIP1B (Agrisera) antibody that cross-reacts with the tobacco IEM localized aquaporin NtAQ1 (Kammerloher *et al.*, 1994) and the thylakoid-specific anti-cytochrome *f* (Agrisera) and anti-D1 (Agrisera) antibodies.

### Gas exchange and carbon isotope discrimination

Measurements of leaf gas exchange alone and those made concurrently with tuneable diode laser spectroscopy to measure carbon isotope discrimination in real time were done as previously described (Pengelly *et al.*, 2012). These measurements were made on the developmentally analogous, young, near-fully expanded leaves from ~40–45 cm-tall glasshouse-grown plants. Mesophyll conductance (*g*_m_, the conductance of CO_2_ diffusion from intercellular airspace to the chloroplast stroma) was calculated as described by Evans and von Caemmerer (2013).

## Results

### Generation and growth of tob^BicA^ plants

Transplastomic tobacco expressing the cyanobacterial bicarbonate transporter BicA from *Synechococcus* PCC7002 were obtained by biolistic bombardment of *N. tabacum* leaves with the plastome-transforming plasmid pRVBicA (Fig. 1A). Spectinomycin-resistant leaf material was passed through three rounds of regeneration on selective media and two independently transplastomic lines, B4 and B7, were identified by Southern blotting (Fig. 1B). As predicted for correct homologous recombination of *bicA* into the plastome, the ^32^P-TGF probe hybridized to a 3.8-kB *Hind*III DNA fragment in both B4 and B7 and a 7.7-kb DNA fragment in wild-type tobacco. The 7.7-kb hybridization signal was not detectable in either B4 or B7 samples, indicating both lines were homoplastomic. Correct integration of *bicA* and *aadA* into the plastome was confirmed by PCR and sequencing (data not shown). When T_0_ plants were transferred to soil and grown to maturity in the greenhouse, no obvious phenotypic differences in growth and development were observed compared to wild-type tobacco. No difference in comparative measures of leaf *bicA* mRNA, BicA protein, leaf gas exchange, measures of CO_2_-assimilation rate changes with varying C_i_, plant growth or phenotype could be detected between the T_1_ to *T*_3_ progenies of either the B4 or B7 lines. Therefore, measurements from T_3_ B7 plants referred to as tob^BicA^ are reported.

### Comparison of cellular BicA, D1, and chlorophyll in tob^BicA^ and cyanobacteria

**Fig. 2.**
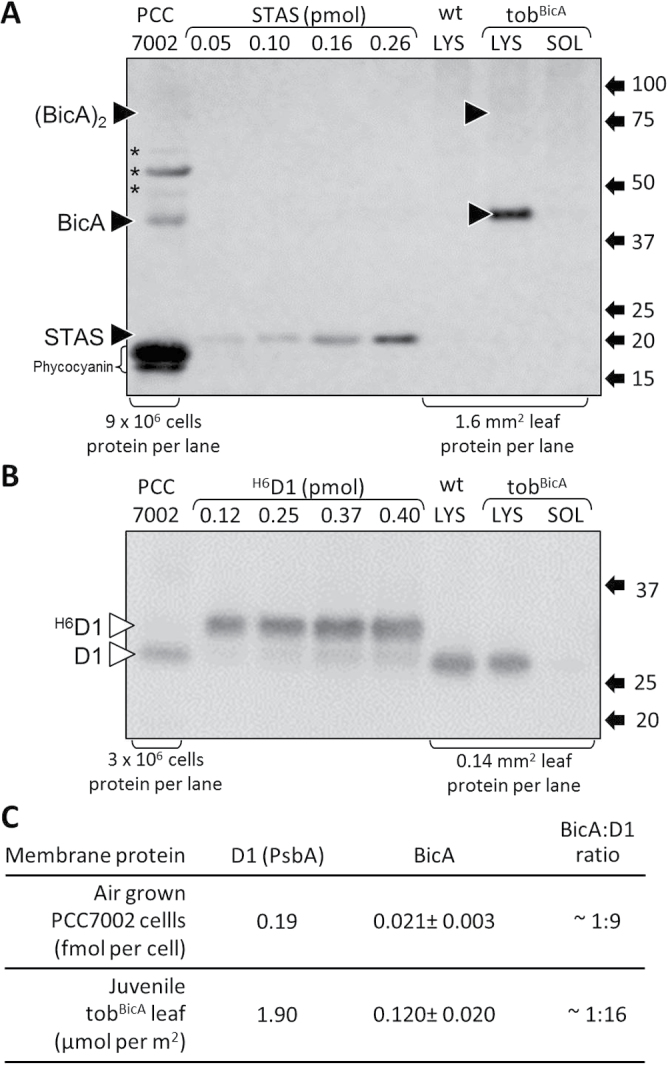
Western blot quantification of BicA and D1. (A and B) SDS PAGE-separated total (LYS) and soluble (SOL) cellular protein extracts from *Synechococcus* PCC7002 cells and a young, upper canopy juvenile tob^BicA^ leaf were blotted onto nitrocellulose and probed with antibodies against BicA (A) and the photosystem II D1 protein (B). The number of PCC7002 cells and the area of leaf from which protein samples were taken are indicated. Expression levels were quantified by coseparation and immunoblotting with known amounts of purified 6×histidine-tagged BicA STAS domain and D1 proteins. Indicated are the positions of the BicA monomer and the (BicA)_2_ dimer (black triangles), the non-BicA proteins in PCC7002 recognized by the BicA antibody (*), the chemifluorescent phycocyanin and the positions and sizes (kDa) of molecular weight protein standards. (C) Comparative levels of D1 and BicA in tob^BicA^ and *Synechococcus* PCC7002 total cellular samples.

Immunoblot detection of BicA in the total protein but not the soluble protein of young, expanding, upper canopy tob^BicA^ leaves supported the prospect that it was membrane associated (Fig. 2A). This was further supported by the detection of both the BicA monomer (separating by SDS PAGE at an apparent mass of ~44kDa) and the less abundant BicA dimer (separating at ~88kDa) in tob^BicA^ leaves, as seen for *Synechococcus* PCC7002 cellular protein (Fig. 2A). BicA levels were quantified against known amounts of the purified 20kDa cytosolic exposed STAS peptide domain of *Synechococcus* PCC7002 BicA (Fig. 2A). The D1 levels in the same samples were quantified using an antibody that equally recognizes plant and cyanobacterial D1 protein (Fig. 2B). The molar content of BicA were similarly 9-fold and 16-fold lower than the amount of D1 in air-grown *Synechococcus* PCC7002 cells and young juvenile tob^BicA^ leaves (Fig. 2C). A comparison of BicA abundance relative to a chloroplast envelope protein was not feasible as no candidates with suitable protein standards needed for quantitative analysis could be sourced.

### Comparative growth and development of leaf biochemistry in wild-type and tob^BicA^ plants.

**Fig. 3.**
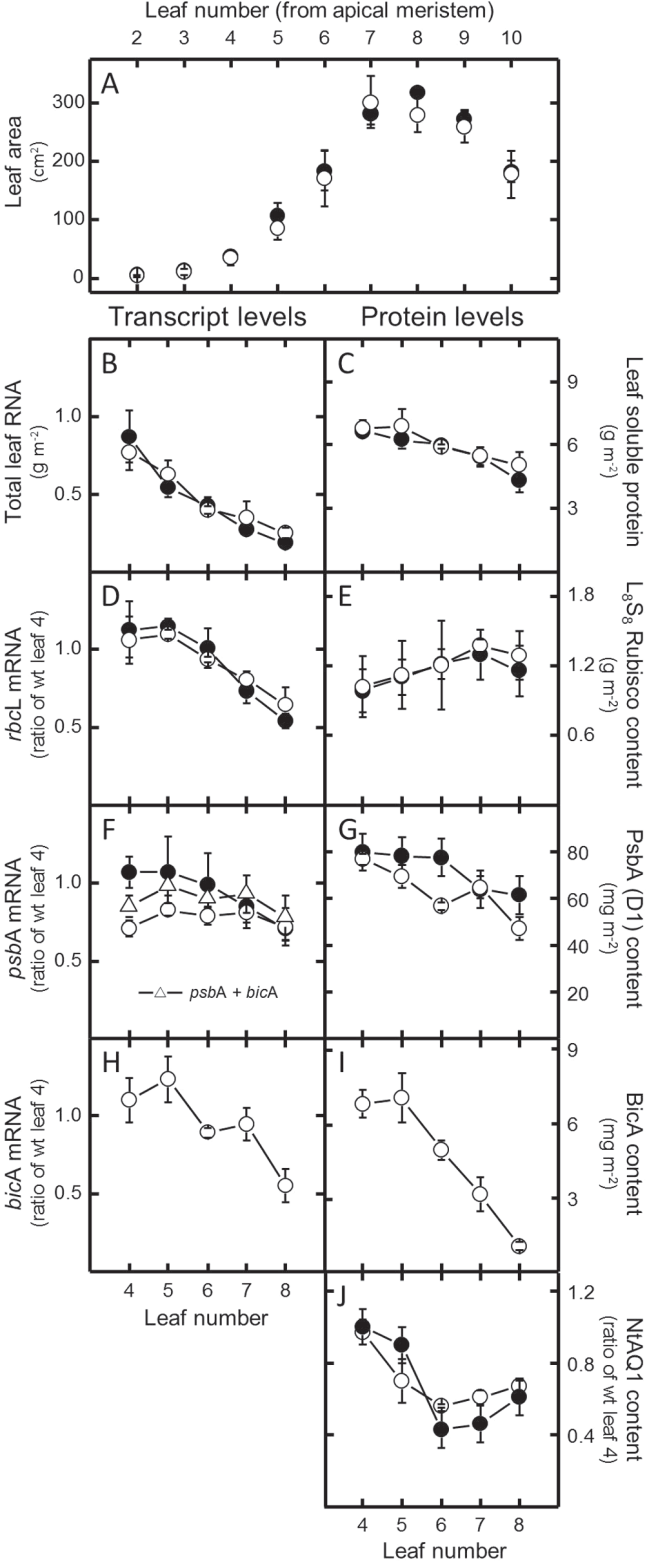
Comparative leaf development and biochemistry of 30-day-old wild-type tobacco (wt) and T_3_ tob^BicA^ plants. Replicate wt ({filled circle}) and tob^BicA^ {open circle} plants were sampled at the same stage of exponential growth (25.0±1.4cm in height). (A) Number and area of wt and tob^BicA^ leaves at the time of sampling. (B–I) Samples from the 4th to 8th upper canopy young leaves were tested for total leaf RNA (B), soluble leaf protein (C), *rbcL* mRNA content (D), amount of Rubisco holoenzyme (L_8_S_8_) (E), *psbA* mRNA pool (including the total pool of *psbA* and *bicA* mRNA (Δ) in tob^BicA^ leaves that share the same promoter/5′-UTR sequence, to which the psbA probe (Fig. 1A) equally hybridizes) (F), D1 (PsbA) leaf content (G), *bicA* mRNA level (H), and changes in the amounts of BicA (I) and NtAQ1 (J). Data are mean±SE of three measurements from Northern and Western blots. See Supplementary Fig. S1 for representative RNA blots used to quantify steady-state mRNA pools.

Consistent with analyses of earlier progeny, the growth, leaf, and canopy phenotype of the T_3_ tob^BicA^ progeny matched that of the wild type (Fig. 3A). Total RNA levels in the upper canopy leaves (leaves 4–8) showed no difference between tob^BicA^ and wild type per unit leaf area (Fig. 3B). Both tobacco genotypes showed decreased total RNA levels during leaf ontogeny with ~6-fold higher concentrations in the upper canopy juvenile leaves relative to the lower newly fully expanded leaves. In contrast, the same leaves only showed a 0.6-fold reduction in their soluble protein contents (Fig. 3C). Similar disparities between transcript and protein levels were found for Rubisco *rbcL* mRNA and L-subunit content measurements, where in both wild type and tob^BicA^ 2-fold reductions in relative *rbcL* transcript abundance down the canopy (Fig. 3D) contrasted with increasing Rubisco amounts of around 30% (Fig. 3E). Conversely, the decrease in *psbA* mRNA per area in the expanding wild-type leaf (Fig. 3F) more closely resembled the decrease in D1 protein down the canopy (Fig. 3G).

A distinguishing feature of the tob^BicA^ leaves was that *psbA* mRNA levels were significantly lower in the upper younger leaves compared to the wild type (Fig. 3F), although largely without affecting steady state D1 content (Fig. 3G). The lower *psbA* transcript abundance in the young tob^BicA^ leaves may be linked to the use of the *psbA* promoter and 5′-UTR and 3′-UTR regulatory sequences to also drive *bicA* expression (Fig. 1A). Indeed, when *psbA* and *bicA* mRNA levels in tob^BicA^ were considered together, their levels more closely aligned with the levels of *psbA* transcript in the wild type (Fig. 3F, triangles). By comparison, however, the steady-state *bicA* mRNA levels were only 10–15% of those of *psbA* (Supplementary Fig. S1 available at *JXB* online) with unmatching reductions in relative abundance during leaf expansion (Fig. 3H) despite sharing the same regulatory sequences. Analogous to the reducing *bicA* mRNA levels down the tob^BicA^ canopy, BicA levels also decreased, albeit at a more striking rate, with the BicA content in newly fully expanded lower leaves (leaf 8) being 8-fold lower than in the young, expanding upper canopy leaves (leaf 4; Fig. 3I). In contrast, only ~2-fold variation in the amount of chloroplast envelope-specific tobacco aquaporin NtAQ1 levels was found in the same tob^BicA^ leaves whose relative amounts matched those measured in the wild type (Fig. 3J).

### Localization of BicA in tob^BicA^ within chloroplasts

**Fig. 4.**
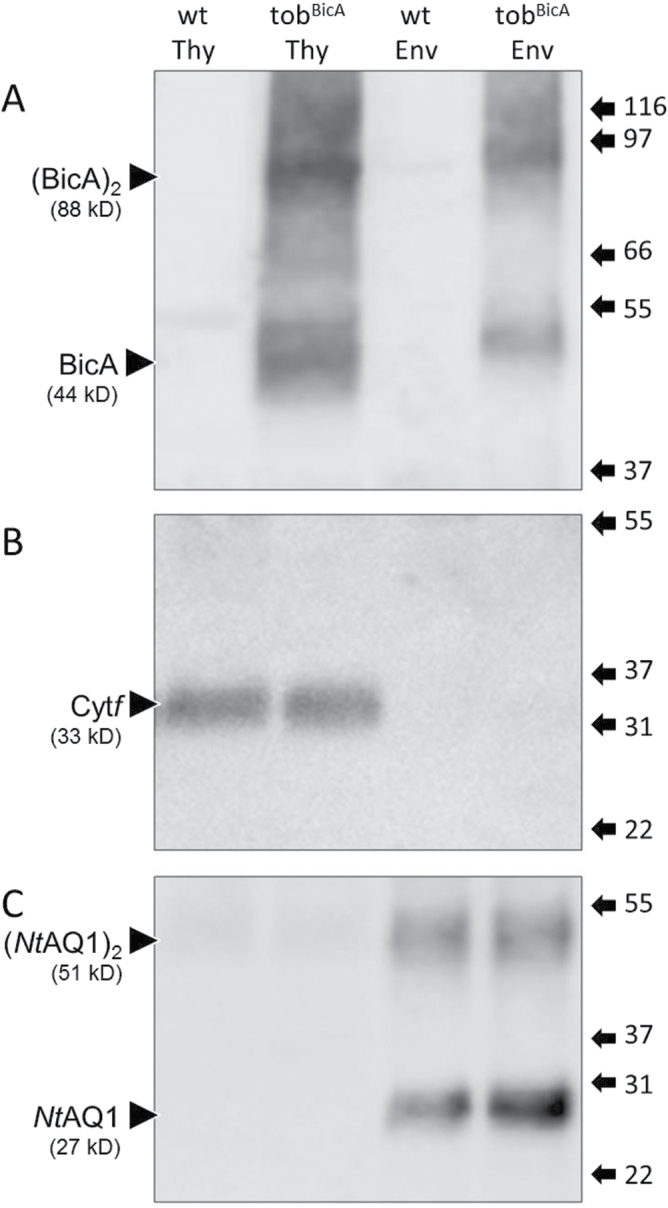
Localization of BicA in the thylakoid and envelope membranes of tobacco chloroplasts. Thylakoid (Thy) and chloroplast envelope (Env) protein fractions were isolated from juvenile wild-type tobacco (wt) and T_3_ tob^BicA^ leaves and separated by SDS-PAGE. Representative Western blots performed with antibodies against BicA (A), the thylakoid-specific cytochrome *f* (Cyt*f*) protein subunit of the cytochrome *b*_6_ *f* complex (B), and the *N. tabacum* envelope-specific aquaporin protein (NtAQ1) (C). Arrows indicate bands corresponding to the BicA protein monomer and (BicA)_2_ dimer. The position and size (in kDa) of molecular weight protein standards are shown.

Immunoblot analyses of chloroplast envelope and thylakoid membrane proteins from replica preparations of Percoll gradient-purified chloroplasts repeatedly showed BicA localized to both membrane fractions, presumably as an integral protein complex in both lipid-bilayer environments (Fig. 4A). The purity of these distinctive membrane fractions was confirmed by probing with antibodies to thylakoid proteins Cyt*f* (Fig. 4B) and D1 (data not shown) as well as the plastid envelope NtAQ1 aquaporin (Fig. 4C). Estimating the relative distribution of BicA in each membrane fraction from immunoblots (such as Fig. 4A) were impractical due to the disproportionate method by which each membrane fractions was purified. However, comparison of the molar BicA to D1 ratio in the 48-h-darkened young leaves used to purify the chloroplasts (1:14) and in the purified thylakoid protein fraction (1:19) (data not shown) indicated approximately 75% of the BicA in tob^BicA^ was located in the thylakoid membranes and the remaining 25% in the plastid envelope. Transmission electron microscopy of wild-type and tob^BicA^ chloroplasts showed the integration of BicA into both membrane fractions had no effect on chloroplast ultrastructure with regard to thylakoid stacking and chloroplast envelope integrity (Supplementary Fig. S2).

### Evaluation of BicA functionality in tob^BicA^ leaves

**Fig. 5.**
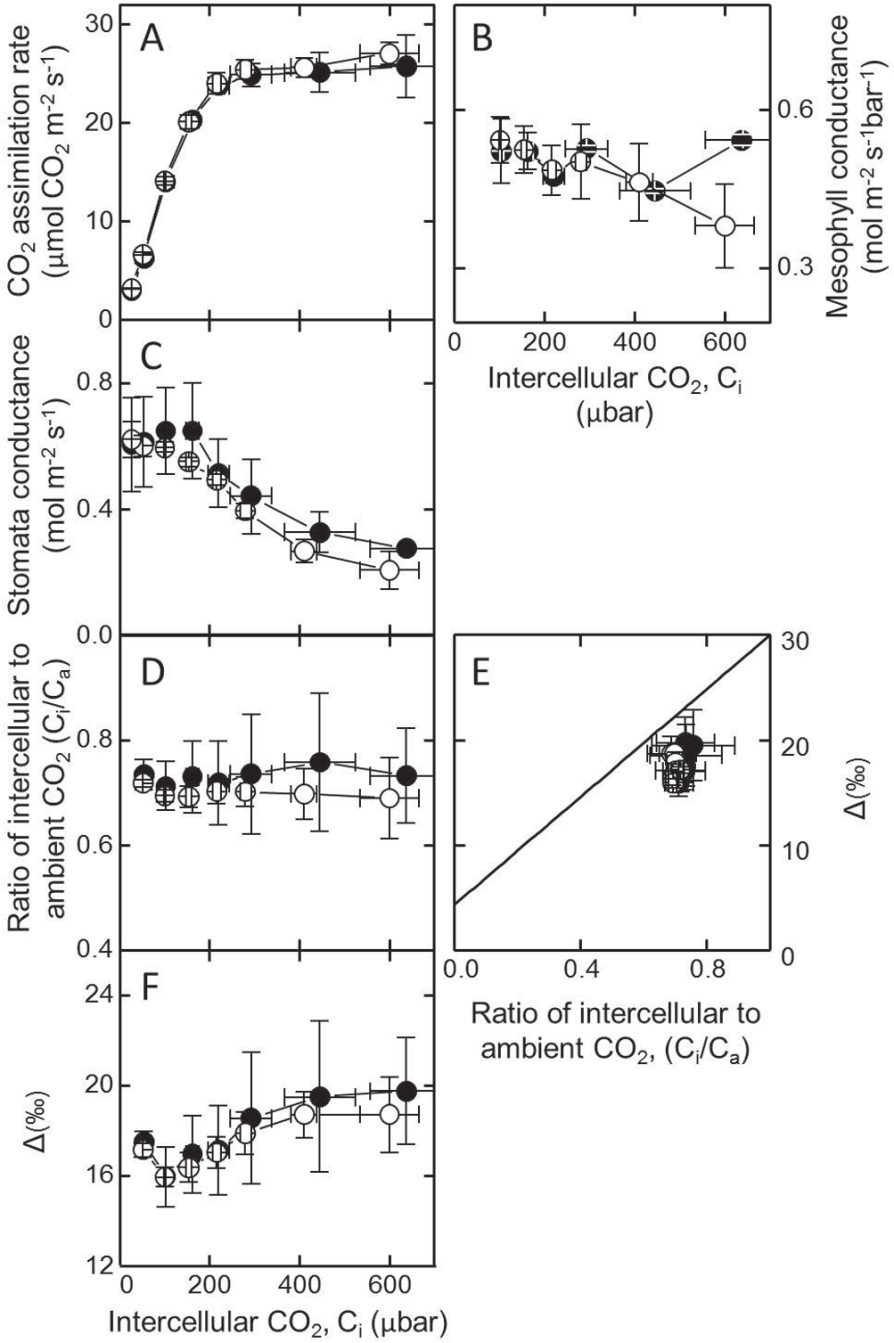
Leaf gas exchange analyses of wild-type tobacco ({filled circle}) and tob^BicA^ {open circle} plants. (A–D) Concurrent measurements from physiologically comparable upper canopy leaves of CO_2_ assimilation rate (A), mesophyll conductance (B), stomatal conductance (C), and ratio of intercellular leaf CO_2_ (C_i_) to ambient CO_2_ (C_a_), as a function of C_i_ (D). (E, F) Variations in carbon isotope discrimination relative to C_i_/C_a_ (E) and C_i_ (F). All measurements were made under 2% O_2_, 1500 μmol quanta m^–2^ s^–1^ illumination and leaf temperature of 25 °C. Data are mean±SE of three experimental replicates.

Leaf gas exchange and carbon isotope discrimination measurements were made to test for evidence of BicA functionality in elevating inorganic carbon (i.e. HCO_3_^–^ and hence CO_2_) levels in the chloroplasts of tob^BicA^. It is predicted that an active CCM in C_3_ chloroplasts may be accompanied by a lower CO_2_-compensation point (Γ; Price *et al.*, 2010). However, the Γ of tob^BicA^ (49.8±0.5 μbar CO_2_, *n*=6 leaves) at ambient oxygen (20.6%, v/v) matched the Γ of the wild type (50.2±1.5 μbar CO_2_, *n*=4 leaves). Likewise the CO_2_-assimilation rates in response to varying CO_2_ pressures were identical for both tob^BicA^ and wild type when tested at ambient O_2_ (data not shown) and 2% O_2_ (Fig. 5A). Stomatal conductance (Fig. 5C) also showed little or no variation between tob^BicA^ and wild type, with both tobacco genotypes showing similar intercellular to atmospheric CO_2_ concentrations (C_i_/C_a_) ratios across differing C_i_ (Fig. 5D). Carbon isotope discrimination was measured at 2% O_2_ to limit possible photorespiratory fractionations (Evans and von Caemmerer, 2013); if BicA was active, reductions in carbon isotope discrimination (Δ) would be expected, due to the known carbon isotope fractionations associated with CO_2_ bicarbonate exchange at equilibrium where ^13^C preferentially accumulates in bicarbonate (Mook *et al.*, 1974). However, no difference in photosynthetic Δ measured in real time during gas exchange relative to C_i_/C_a_ (Fig. 5E) or in response to varying C_i_ (Fig. 5F) were evident between wild-type tobacco or tob^BicA^, indicating no evidence of elevated inorganic carbon in the tob^BicA^ chloroplasts. Accordingly, mesophyll conductance (Fig. 5B) calculated from the carbon isotope discrimination measurements was similar for the two genotypes.

## Discussion

Expressing active bicarbonate (HCO_3_^–^) transporters in the chloroplast membrane forms the primary step in bioengineering strategies to reduce Rubisco oxygenation in plants undertaking C_3_ photosynthesis and improve their photosynthetic capacity (Price *et al.*, 2013). The current study shows that the *Synechococcus* PCC7002 Na^+^-dependent HCO_3_^–^-transporter protein BicA can be successfully integrated into tobacco chloroplast membranes by integration of *bicA* into the plastome. Compared with BicA levels in cyanobacteria (one BicA per nine D1 subunits), comparable levels of BicA were produced in the tob^BicA^ leaves (as much as one BicA per 16 D1), albeit with the majority of the BicA (approximately 75% in juvenile developing leaves) integrating into the thylakoid membranes rather than the chloroplast IEM (Fig. 4). Correct assembly of BicA complexes in tob^BicA^ leaves seems likely since immunoblot analyses identified BicA oligomeric assemblies similar to those found in PCC7002 cells (Fig. 2A), particularly in the more concentrated tob^BicA^ chloroplast membrane protein preparations (Fig. 4A). Physiological analyses, however, showed no evidence of bicarbonate transporter activity in tob^BicA^ leaves (Fig. 5). Together, these findings ratify the versatility of plastome transformation as a practical approach for integrating bicarbonate transporters into plant chloroplasts and highlight future challenges to identify solutions that enable cyanobacterial transporter protein functionality and specific IEM integration in plant plastids.

The findings that BicA incorporated into both the chloroplast envelope and thylakoid membranes contrasts with the transplastomic expression of the *Arabidopsis* IEM Tic40 protein (AtTic40) that was shown to specifically integrate into the IEM of tobacco chloroplasts, albeit without evidence of functionality (Singh *et al.*, 2008). Unlike the tob^BicA^ plants, the levels of AtTic40 produced in the tobacco plastids were in massive excess of requirement and resulted in abnormal chloroplast development and slowed plant growth. A transplastomic approach in tobacco has also shown successful integration of a functional *Chlamydomonas reinhardtii* plastid terminal oxidase (CrPTO) into tobacco plastid thylakoids, although without examining the possible cointegration of the CrPTO into the plastid IEM (Ahmad *et al.*, 2012). It is interesting to speculate on the effect that a functional BicA wrongly targeted to the thylakoid would have on chloroplast performance. A possible consequence might be reduction of the luminal ΔpH gradient and ATP supply resulting from the conversion of HCO_3_^–^ to CO_2_. However, this could also result in increased stromal CO_2_ levels that would benefit Rubisco catalysis.

The molecular signatures that enable targeted integration of leaf proteins into the outer and inner plastid membranes and then to achieve their correct folding topology remain challenging questions in cell biology. Similar to AtTic40 and CrPTO, this study relied on inherent topology-determining sequence information within PCC7002 BicA to correctly integrate it into the IEM of tobacco chloroplasts. These molecular signatures in BicA likely entail hydrophobic domains and the location of positive charges on the cytoplasmic and stromal sides of the protein (Shelden *et al.*, 2010). For chloroplast membrane proteins, particular focus has been on deciphering the mechanistic details of the two targeting pathways for incorporating nuclear-encoded proteins into the IEM. The stop-transfer pathway involves protein passage through the translocon outer complex then passage into the lipid bilayer (Schunemann, 2007) while the post-import (or conservative sorting) pathway sees membrane-targeted proteins pass into the stroma prior to integrating into the IEM (Jarvis and Robinson, 2004; Tripp *et al.*, 2007; Viana *et al.*, 2010). Possible sequence determinants involved in post-import targeting of membrane proteins into the IEM hypothesize involvement of transmembrane domain sequences that help in obtaining correct topology (Viana *et al.*, 2010) and serine/proline sequences that help avoid stop-transfer insertion (Tripp *et al.*, 2007). The effectiveness of endogenous chloroplast post-import signal sequences to target recombinant membrane proteins, such as those from cyanobacteria, relies both on the inherent topology forming features of the recombinant protein and the context of the signal sequences.

The chloroplast-made IEM-localized 27kDa CemA protein of higher plant plastids (Sasaki *et al.*, 1993) poses a potential target for identifying its post-translational IEM signal sequences. For example, targeted changes to the 20 amino acid N-terminal transmembrane helices predicted for CemA (residues 6–25), or its chimeric integration into another membrane protein such as BicA, may help to identify whether this domain functions as an IEM signal sequence. This transgenic approach is advantaged by the homologous recombination route by which plastome *cem*A, or mutant chimeric variants of the gene, can be genetically modified with pinpoint accuracy. Given the role of CemA homologues in indirectly influencing inorganic carbon uptake via regulation of proton extrusion in *Chlamydomonas* (Rolland *et al.*, 1997) and cyanobacteria as PxcA (Sonoda *et al.*, 1998), transgenic modification to CemA activity in tobacco chloroplasts and analysing the carbon isotope discrimination and photosynthetic physiology of these genotypes, as undertaken with tob^BicA^ (Fig. 5), pose one approach for validating the role for CemA in leaf chloroplasts.

In recent years, the IctB membrane protein from cyanobacteria has been a popular target for integrating into plant plastids. Initial assertions that IctB constituted a bicarbonate transporter protein have since been discounted (Shibata *et al.*, 2002). However, growth benefits have been seen in C_3_ plants when *ictB* is fused with N-terminal plastid transit sequence and then transformed into the host genome, albeit without confirmation of IctB protein synthesis or cellular localization (Lieman-Hurwitz *et al.*, 2003). Such measurements are necessary to establish a mechanistic link between transgene expression and observed phenotype with any certainty. This reinforces the view that experimental rigor is needed in analysing the cellular localization and functionality of recombinant membrane proteins, particularly those targeted to leaf plastids, as has been a demonstrated objective of this study.

An ongoing challenge of plastome transformation in being able to predictably express recombinant proteins at desired levels (De Marchis *et al.*, 2012; Scotti *et al.*, 2012; Hanson *et al.*, 2013). Despite sharing analogous *psbA* regulatory genetic sequences, the excessive levels of AtTic40 produced in tobacco plastids (~15% of leaf protein (Singh *et al.*, 2008) contrasted with levels of BicA produced in tob^BicA^ leaves, which accounted for, at most, ~0.1% (w/w) of the protein content early in leaf ontogeny (Fig. 3). Relative to the high levels of the thylakoid photosystem II D1 protein, the molar abundance of BicA was ~8–10% that of D1 (assuming 75% thylakoid localization), which, in turn, mimicked the 9:1 molar ratio of D1:BicA in *Synechococcus* PCC7002. This suggests that the chloroplast BicA levels in tob^BicA^ are likely suitable for adequate levels of inorganic carbon pump function. Comparing BicA abundance against the levels of native chloroplast IEM proteins (such as proteins in the TIC complex or NtAQ1; Fig. 4C) were precluded by experimental limitations with regard to availability of suitable protein standards necessary for quantitative analysis. Similarly, limitations in isolating intact chloroplasts from mature tobacco leaves precluded analysis of whether BicA distribution between chloroplast thylakoid membranes and IEM changes during leaf ontogeny. The initial cotargeting of plasma membrane-targeted proteins to the thylakoids during development, followed by probable sorting to the plasma membrane via thylakoid–envelope contact points, has been observed previously in cyanobacteria (Pisareva *et al.*, 2011). Possibly, the recycling of thylakoid-located BicA or increased partitioning of thylakoid-located BicA to the IEM during leaf ontogeny might contribute to lower BicA levels in older leaves (Fig. 3I). Notably, this almost linear reduction in BicA content with leaf age was not shared by Rubisco, D1, or NtAQ1 levels, where more modest changes in content were observed within the upper canopy leaves.

These results show that strong, stable targeting of BicA to the IEM was clearly achieved, despite significant amounts present in the thylakoids, but critically without any detectable negative effects on plant growth or photosynthetic performance. However, the results also highlight that identifying solutions for activating heterologous HCO_3_^–^-transporter activity in plant chloroplasts poses another key challenge. The need to understand activation of bicarbonate transporters appears to be a consequence of the fact that high-flux transporters such as BicA need to be dark inactive in cyanobacteria to avoid futile cycling and wastage of metabolic energy, but then reactivated in the light (Price *et al.*, 2008; Price and Howitt, 2011). One possibility was that some of the general signals in the illuminated chloroplast (e.g. thioredoxin status, IEM gradients for protons, sodium and membrane potential) would have allowed BicA activation, but this appears not to be the case. Ongoing efforts are strongly aimed at discovering the basis of BicA activation in its native environment in cyanobacterial cells with the ultimate goal to be able to re-establish correct regulation of bicarbonate transporter activity in the chloroplast envelope of higher plants. This involves investigations into many aspects of possible BicA regulation by post-translational modifications and/or protein–protein interactions (Price *et al.*, 2013). Once the minimum requirements for controlling BicA function in heterologous systems have been identified, plastome and/or nuclear transformation can be employed to advance to the next level of enabling BicA function in these tob^BicA^ plants.

In conclusion, the physiologically relevant level of stable BicA expression achieved in the chloroplast envelope in tob^BicA^ leaves poses a pioneering step towards introducing active bicarbonate uptake into leaf chloroplasts and the ensuing establishment of a physiological CCM into C_3_ plants. This work demonstrates a rigorous and comprehensive experimental strategy to generate, genetically and biochemically validate, and phenotypically characterize transplastomic tobacco plants for potential alterations in photosynthetic function and growth. These methodologies set out a valuable framework for future manipulations and experimental examination of alterations in photosynthesis and chloroplast function.

## Supplementary material

Supplementary data are available at *JXB* online.

Supplementary Fig. S1. Representative RNA (Northern) and protein (Western) blots of steady state mRNA pools and their translated protein products from the same area of comparable leaves from tob^BicA^ and wild-type tobacco.

Supplementary Fig. S2. Transmission electron micrographs showing the comparable chloroplast ultrastructure in leaves from tob^BicA^ and wild-type tobacco.

Supplementary Data
