# Supplementary material for: Transplastomic integration of a cyanobacterial bicarbonate transporter into tobacco chloroplasts
Source: J Exp Bot. 2014 Apr 18;65(12):3071–80. doi: 10.1093/jxb/eru156 (PMC4071830; doi:10.1093/jxb/eru156)
Supplement: Supplementary Data [file supp_65_12_3071__index.html]

Transplastomic integration of a cyanobacterial bicarbonate transporter into tobacco chloroplasts — Transplastomic integration of a cyanobacterial bicarbonate transporter into tobacco chloroplasts — Supplementary Data 

# Transplastomic integration of a cyanobacterial bicarbonate transporter into tobacco chloroplasts

## Supplementary Data

Data files

**Files in this Data Supplement:**

- Supplementary Data - Supplementary Data
