# Supplementary material for: Transplastomic integration of a cyanobacterial bicarbonate transporter into tobacco chloroplasts
Source: J Exp Bot. 2014 Apr 18;65(12):3071–80. doi: 10.1093/jxb/eru156 (PMC4071830; doi:10.1093/jxb/eru156)

Transplastomic integration of a cyanobacteria bicarbonate transporter into tobacco chloroplasts. *JJL Pengelly, B Forster, S von Caemmerer, MR Badger, GD Price, SM Whitney*

SUPPLEMENTARY DATA

Supplementary figure S1.

Representative RNA (Northern) and protein (Western) blots of steady state mRNA pools and their translated protein products from the same area of leaf taken from comparable positions in the upper canopy of 25.0 ± 1.4 cm in height wild-type tobacco and tob^{BicA} plants (lanes represent leaves 4-8 from the apical meristem). Data from such analyses were used to quantify the leaf mRNA steady state levels and the corresponding amounts of D1, NtAQ1 and BicA shown in Fig. 3. The detected levels of Rubisco L-subunit (LSU) were used to confirm the amount of leaf Rubisco L_8S_8 holoenzyme quantified by [^{14}C]-CABP binding.

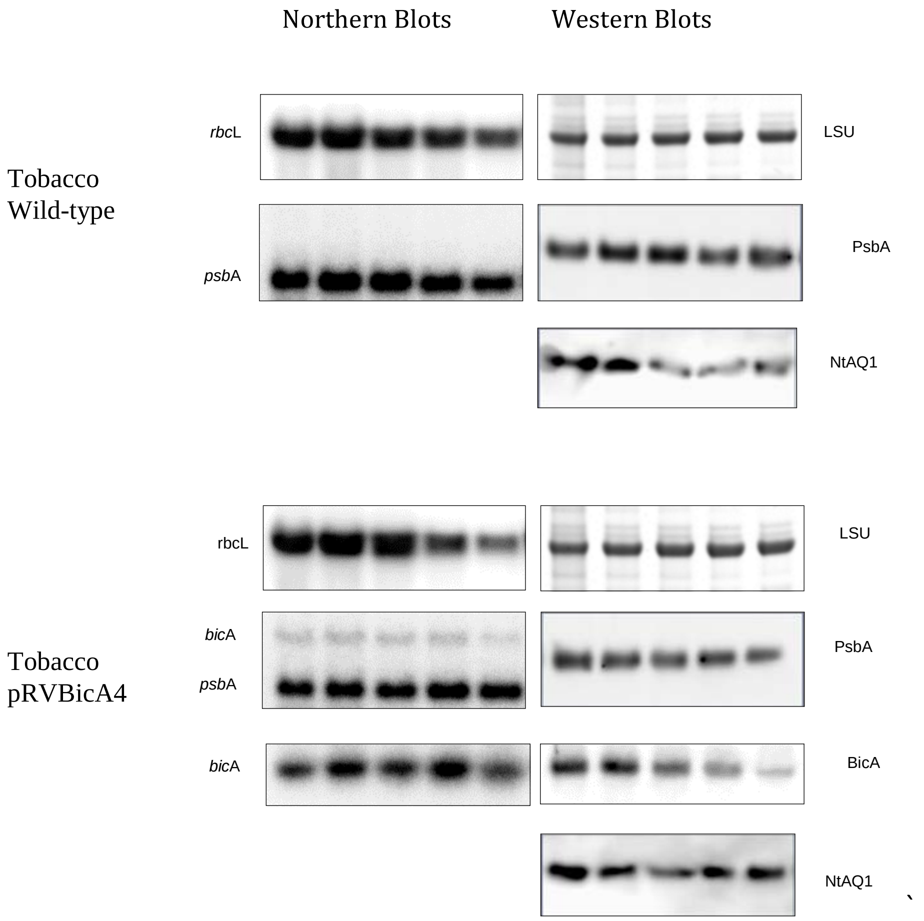

Supplementary figure S2.

Transmission electron microscope images showing the comparable chloroplast ultrastructure in leaves of tob^{BicA} (A, B) and wild-type tobacco (C, D).

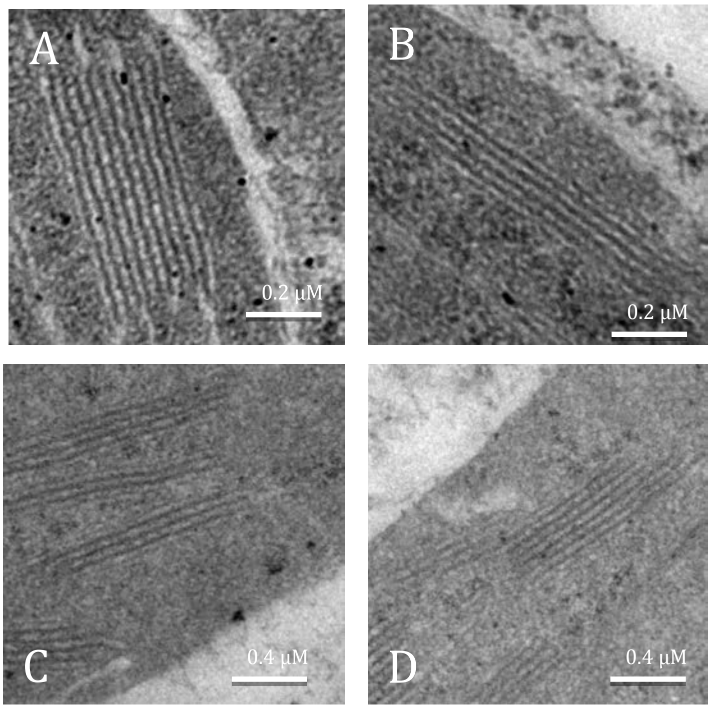

Supplement: Supplementary Data [file supp_eru156_jexbot119446_file001.pdf]
